# Role of ECG in the Accidental Finding of an Atrioventricular Septal Defect in an Asymptomatic Patient Undergoing Cosmetic Surgery

**DOI:** 10.7759/cureus.52406

**Published:** 2024-01-16

**Authors:** Pramod Bhattarai, Monika Karki

**Affiliations:** 1 Pulmonary Medicine, Howard University Hospital, Washington, DC, USA; 2 Critical Care Medicine, Larkin Community Hospital Palm Springs Campus, Hialeah, USA; 3 Internal Medicine, Harlem Hospital Center, New York City, USA; 4 Cardiovascular Disease, Broward Health Medical Center, Fort Lauderdale, USA

**Keywords:** ventricular septal defect (vsd), cardio thoracic surgery, primum asd, adult congenital heart disease (achd), atrioventricular septal defect (avsd)

## Abstract

Electrocardiogram (ECG) is an important diagnostic tool in identifying congenital heart disease (CHD), as demonstrated by this case of a 48-year-old female who presented for a preoperative evaluation for cosmetic surgery. ECG showed a right bundle branch block (RBBB) and first-degree atrioventricular (AV) block, and further testing revealed a primum atrial septal defect (ASD) with mitral valve anterior leaflet cleft and a membranous ventricular septal defect (VSD). She underwent successful surgical repair and was discharged home without complications. This case highlights the importance of performing additional tests like echocardiography or other imaging modalities in cases of abnormal ECG findings to accurately diagnose the underlying heart condition and ensure proper treatment.

## Introduction

In 1982, Robert Anderson and Anton Becker published a seminal paper titled “Atrioventricular septal defects: What's in a name", which introduced a new classification system for a group of congenital heart diseases (CHDs), while working with heart specimens in Amsterdam [1,2]. Prior to this landmark publication, these congenital conditions were referred to as “atrioventricular canal malformation” or "endocardial cushion defect" [1,2]. Adults living with CHD in the United States are estimated to be about 1.4 million. Atrioventricular septal defects (AVSDs) account for about 7%-17% of all CHD cases, while the prevalence of atrial septal defects (ASDs) is 0.88/1000 adults [3,4]. The clinical presentation of CHD can vary widely among patients. Some individuals may be asymptomatic with the condition being incidentally detected as seen in our patient. In contrast, others may experience symptoms related to heart failure or arrhythmias, depending on its severity. The location, severity, and direction of the shunting lesion play a crucial role in the clinical presentation and heart dilation. There are many complexities associated with shunting and heart dilatation; proximal shunts occur above the tricuspid valve and cause dilation of the right heart while distal shunts occur below the tricuspid valve resulting in left heart dilation [3].

## Case presentation

**Figure 1:**
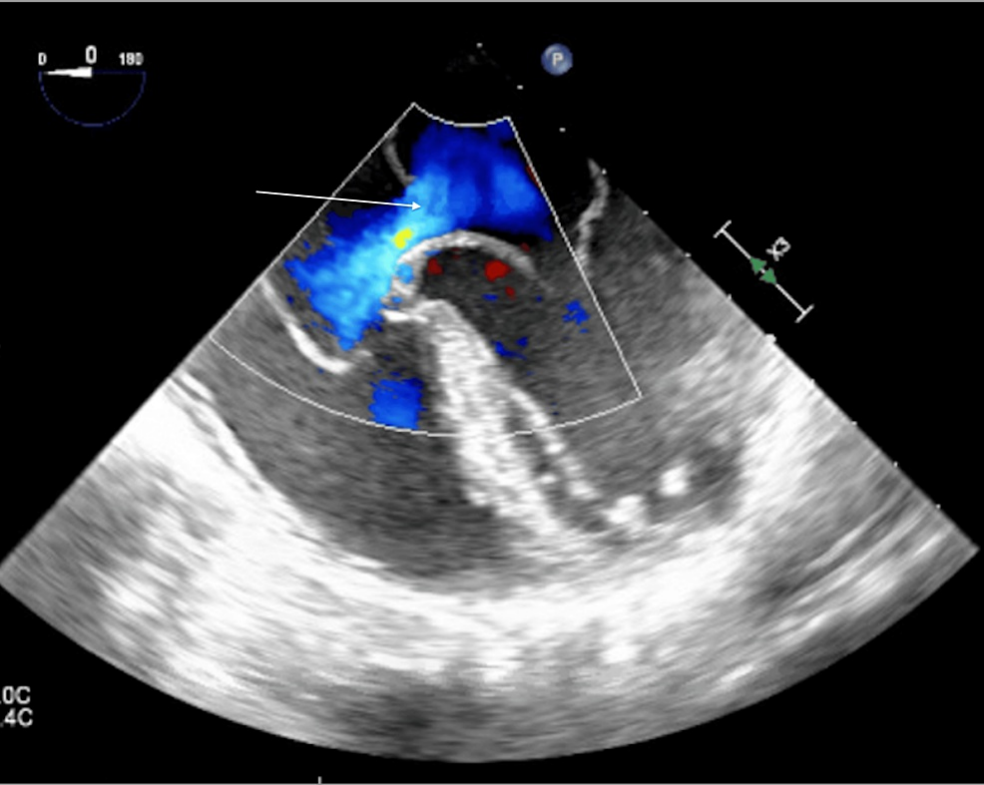
Intraoperative TEE showing the atrial septal defect TEE, transesophageal echocardiography

A 48-year-old female with a past medical history of bilateral breast prosthetic implants was referred to the cardiology department for preoperative evaluation before undergoing cosmetic surgery for breast augmentation due to abnormal findings on her ECG: sinus rhythm with right bundle branch block (RBBB), left anterior fascicular block, and first-degree AV block (Figure 1). She denied any prior cardiac history, cardiac surgery, or symptoms such as chest pain, shortness of breath, orthopnea, dyspnea on exertion, exercise intolerance, palpitations, presyncope, lightheadedness, or syncope. She was physically active and exercised on a regular basis. Physical examinations were unremarkable. A transthoracic echocardiography (TTE) illustrated an ASD, and cardiac magnetic resonance imaging (MRI), which was performed to evaluate anatomy, confirmed the presence of an ostium primum ASD, along with mild dilation of the bilateral atria and the left ventricle, moderate dilation of the right ventricle and an elevated pulmonary to systemic shunt (Qp/Qs) ratio of 1.7:1. Diagnostic cardiac catheterization revealed normal coronary arteries, pulmonary artery pressure, right- and left-sided filling pressure, but an elevated Qp/Qs ratio at 1.7:1 (Table 1).

**Table 1:** Right heart catheterization (RHC) RA; right atrium; RV, right ventricle; PA, pulmonary artery; PCW, pulmonary capillary wedge pressure; Ao, aorta; SVC, superior vena cava; CO, cardiac output; CI, cardiac index; PVR, pulmonary vascular resistance

RHC hemodynamics	
RA a/v/m (mmHg)	11/10/7
RV s/d/e (mmHg)	32/10/13
PA s/d/m (mmHg)	30/10/18
PCW a/v/m (mmHg)	15/18/12
Ao s/d/m (mmHg)	124/76/98
Oxygen saturation (%)	
SVC	70
RA	79
RV	84
PA	82
Ao	95
Fick's CO (L/min)	6.1
Fick's CI (L/min/m^2^)	3.5
PVR (WU)	0.98
Qp:Qs	1.7:1

**Figure 2:**
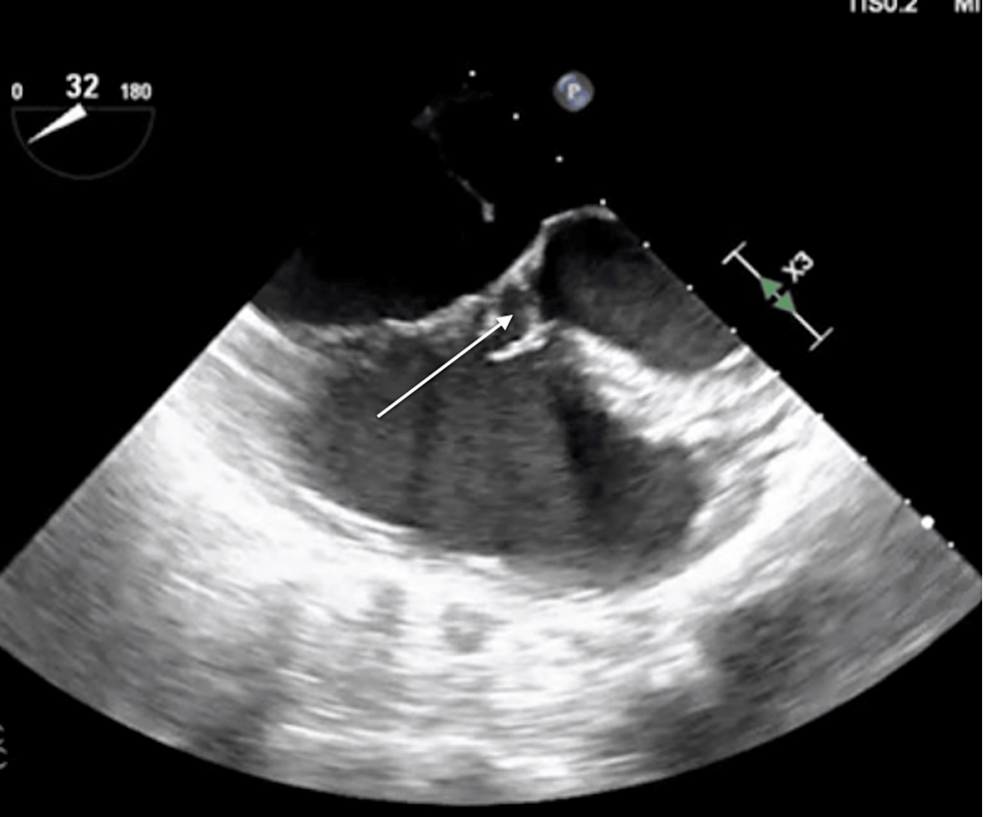
TEE mid-esophageal view showing the membranous ventricular septal defect TEE, transesophageal echocardiography

**Figure 3:**
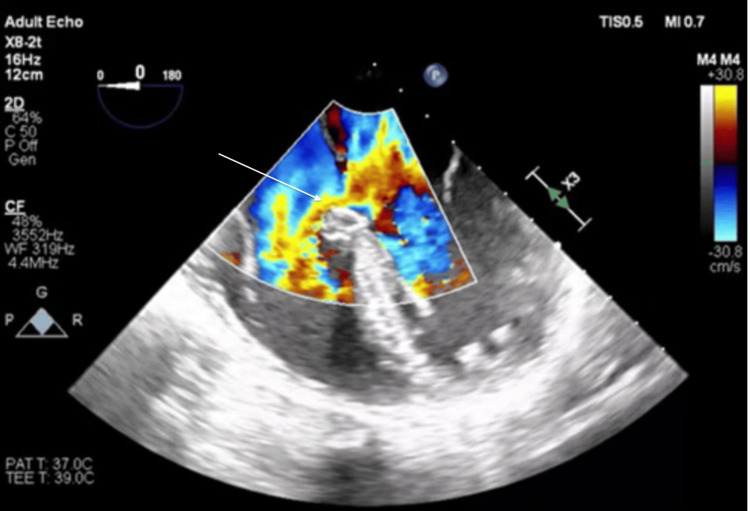
TEE mid-esophageal view with color Doppler showing the atrioventricular septal defect TEE, transesophageal echocardiography

Surgical repair of the primum ASD was recommended and she was taken to the operating room. The intraoperative transesophageal echocardiogram further confirmed the presence of the ostium primum ASD (Figure 2), as well as additional cardiac anomalies including a cleft in the anterior leaflet of the mitral valve and membranous ventricular septal defect (VSD) (Figure 3). The patient successfully underwent repair of the primum ASD and VSD using cardiopulmonary bypass, employing patch repair and valvuloplasty with a mitral valve ring. Following the procedure, the patient was transferred to the intensive care unit; her hospital course was uneventful and and she was discharged a few days later.

## Discussion

AVSD was first defined by Anderson and Becker in 1982 [1]. It is a congenital heart defect and occurs when the endocardial cushions, vital structures responsible for the atrial and ventricular formation, fail to fuse during the first four weeks of embryonic growth [1,2]. This failure leads to the widening of atrioventricular septum, resulting in AVSD [5,6]. Genetic mutations are associated with AVSD, and it often coexists with syndromes such as CHARGE (coloboma, heart defects, atresia of the choanae, retardation of growth, genital underdevelopment, ear abnormalities), Down syndrome, tetralogy of Fallot, etc. These syndromes share common pathogenetic features, including abnormalities in Hedgehog signaling and cilia [5,7]. AVSD can be classified into different types based on the extent of the defect. The four basic components of AVSD include the inlet VSD, primum ASD, cleft mitral valve, and widened anteroseptal tricuspid commissure. If all four components are present, it is characterized as a complete AVSD and if the VSD is absent, a partial AVSD is diagnosed [8,9].

AVSD can remain asymptomatic, making it challenging to diagnose without thorough evaluation. However, symptomatic cases may present with heart failure, arrhythmias, and significant pulmonary hypertension symptoms unless complete repair is performed [9]. The presentation of AVSD in the middle-aged or elderly is relatively rare, and when it occurs, it is often associated with a large left-to-right shunt, indicated by a Qp:Qs ratio greater than 2:1. A retrospective study by Hynes et al. revealed that symptomatic patients with AVSD had left-to-right shunting with a mean Qp:Qs ratio around 3:1. These patients also demonstrated evidence of right ventricular volume overload and elevated pulmonary vascular resistance (PVR) [10].

Accurate diagnosis and evaluation of CHD, including AVSD, rely on various diagnostic tools such as ECG, TTE, and cardiac MRI. ECG provides information on the heart's electrical activity and helps identify arrhythmias. In the context of an AVSD, ECG may reveal characteristic findings such as sinus rhythm with RBBB, left anterior fascicular block, or first-degree AV block. TTE utilizes ultrasound waves to produce images of the heart’s structure and function. It plays a crucial role in diagnosing and assessing the severity of the AVSD, can accurately visualize the ASD, assess the size of the defect, and evaluate the functioning of the valves [11]. Cardiac MRI is more detailed and provides high-resolution images of the heart, blood vessels, and surrounding tissue, offering detailed anatomical and functional information. It is particularly useful in evaluating the anatomy and size of the AV septal defect in AVSD cases [3]. Catheterization is recommended in certain AVSD cases, especially for patients with signs of elevated pulmonary artery pressure or as a preoperative evaluation before surgical correction as it allows for the direct measurement of PVR and helps determine the hemodynamic status of the patient [3].

Surgical closure plays an important role in the management of AVSD. It is required for primum ASD and is reasonable in asymptomatic patients with right heart enlargement. In adults with AVSDs, indication for surgical closure includes a net left-to-right shunt (Qp:Qs ≥ 1.5:1), a pulmonary artery systolic pressure (PASP) of less than 50% systemic pressure, and PVR of less than 1/3 systemic resistance [10]. An untreated AVSD can result in significant long-term mortality rates. Without surgical intervention, only 25% of patients with an AVSD survive beyond the age of 40 and partial AVSD patients over the age of 50 have an estimated annual mortality rate of approximately 7.5% [11]. Thus, timely surgical intervention is crucial for managing AVSD and improving patient outcomes.

## Conclusions

This case report emphasizes the importance of thorough preoperative evaluation, including the recognition of abnormal ECG findings, in patients undergoing surgery, including cosmetic surgeries. Abnormal ECG findings should prompt further evaluation, including cardiac imaging, to determine the extent of the underlying heart conditions and guide treatment decisions. Early diagnosis allows for timely intervention including surgical management, potentially preventing left ventricle remodeling and contractile dysfunction, and can have favorable long-term survival outcomes in patients with CHD. Therefore, it is crucial for healthcare providers to be aware of the potential CHD in patients with abnormal ECG findings and take appropriate steps for further evaluation and treatment.
